# Supplementary material for: Listeria monocytogenes cell-to-cell spread bypasses nutrient limitation for replicating intracellular bacteria
Source: bioRxiv. 2025 Feb 1:2025.01.31.635960. Preprint. [Version 1] doi: 10.1101/2025.01.31.635960 (PMC11838505; doi:10.1101/2025.01.31.635960)
Supplement: 1 [file NIHPP2025.01.31.635960V1-supplement-1.pdf]

bioRxiv preprint doi: https://doi.org/10.1101/2025.01.31.635960; this version posted February 1, 2025. The copyright holder for this preprint (which was not certified by peer review) is the author/funder, who has granted bioRxiv a license to display the preprint in perpetuity. It is made available under aCC-BY 4.0 International license.

SUPPLEMENTAL FIGURES:

Supplemental Figure 1: Growth rate μ and inflection time T_i for intracellular replication of *L. monocytogenes* can be determined directly from measurements of bacterial fluorescence intensity over time.

A. Correlation between number of bacteria in a focus and fluorescence intensity at 6 hours post infection. 62 foci were imaged for this analysis. **B.** Fluorescence intensity plotted as a function of time for a representative bacterial focus imaged from 5 h.p.i. to 15 h.p.i. Open circles represent individual fluorescence intensity measurements, while colored lines depict the exponential fit to the underlying curve at one hour time intervals. **C.** Growth rate plotted as a function of time at one hour time intervals for the bacterial growth curve shown in B. Each data point represents the growth rate calculated from the corresponding exponential fit in B, where the color of each data point in C. matches the color of its respective fit line in B. **D.** Growth rate μ calculated by fitting an exponential function to only the subset of a representative bacterial growth curve from the beginning of the measurement, where integrated time or t_i = 5 h.p.i., to the time corresponding to the value of the x-axis at each data point. Three specific points on the curve are highlighted in salmon, red and maroon and depict growth constants obtained using fits from t_i = 5 h.p.i. to t_i = 7.1 h.p.i., t_i = 9.6 h.p.i. And t_i = 15 h.p.i. respectively. E. - G. Remaining graphs show the plot of fluorescence intensity over time for the length of the experiment (blue curve) against an exponential fit to all data points collected from (E) 5 h.p.i. to 7.1 h.p.i. (salmon dashed line), (F) 9.6 h.p.i. (red dashed line) or (G) 15 h.p.i. (maroon dashed line).

bioRxiv preprint doi: https://doi.org/10.1101/2025.01.31.635960; this version posted February 1, 2025. The copyright holder for this preprint (which was not certified by peer review) is the author/funder, who has granted bioRxiv a license to display the preprint in perpetuity. It is made available under aCC-BY 4.0 International license.

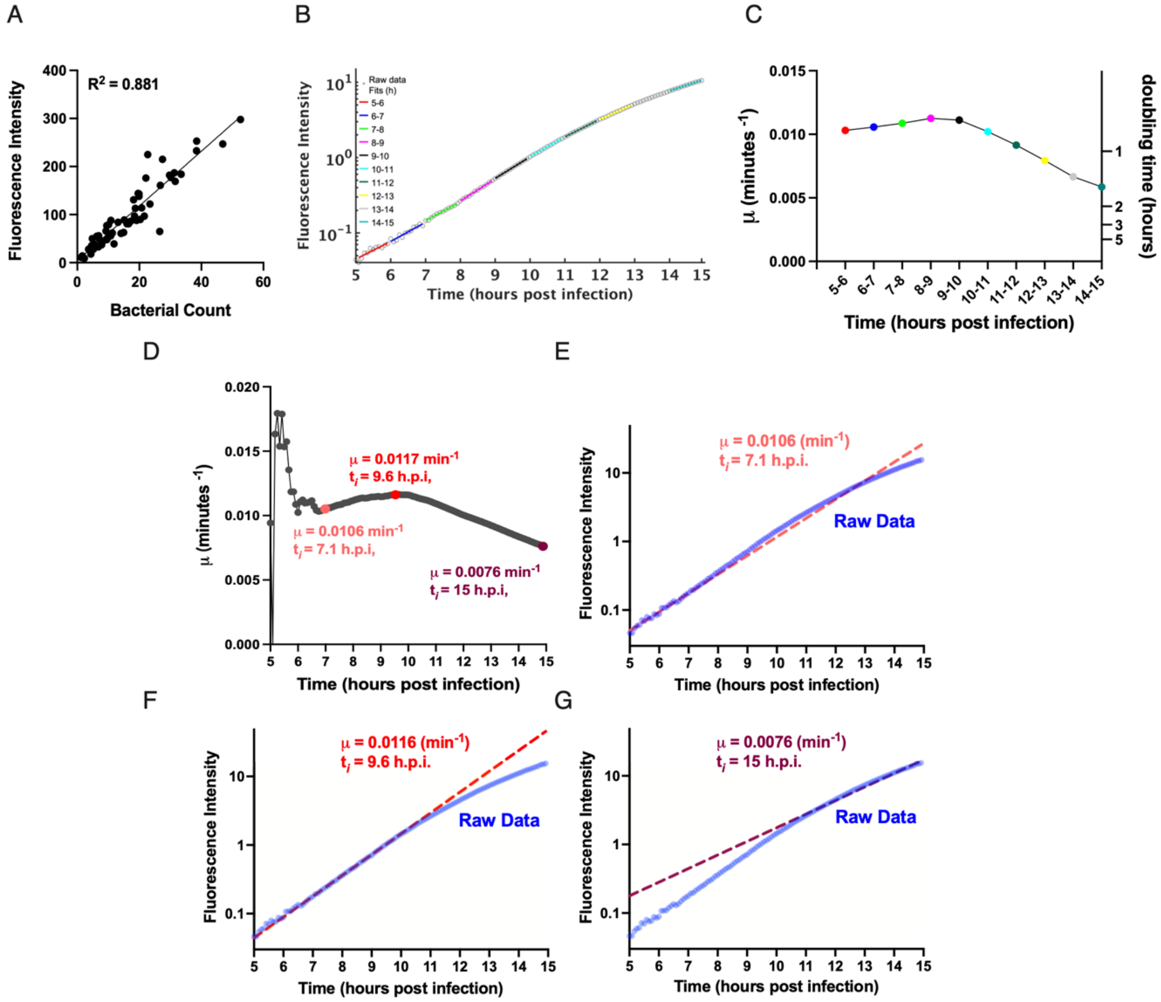

Supplemental Figure 1: Growth rate μ and inflection time T_i for intracellular replication of *L. monocytogenes* can be determined directly from measurements of bacterial fluorescence intensity over time.

bioRxiv preprint doi: https://doi.org/10.1101/2025.01.31.635960; this version posted February 1, 2025. The copyright holder for this preprint (which was not certified by peer review) is the author/funder, who has granted bioRxiv a license to display the preprint in perpetuity. It is made available under aCC-BY 4.0 International license.

Supplemental Figure 2: Stochastic simulations of *L. monocytogenes* cell-to-cell spread aid in determining inflection point for distinct regions of a bacterial focus.

A. To partition a focus into 5 regions, alpha shapes were first generated to bound the maximum intensity projection of binarized bacteria from the beginning of the time lapse recording to time points set 2 hours apart, such as 7 and 9 hours post infection. The difference between two consecutive projections yielded a region within which bacterial growth could be quantified. **B**. Stochastic simulations of bacterial spread (Ortega et al., 2021) were used to generate realistic simulated foci matching the average growth characteristics of the experimental data, and were analyzed using the same methods. Bacterial replication was set to occur at a constant rate for the duration of each simulation. Quantification of average bacterial fluorescence intensity is plotted as a function of time within distinct regions of a simulated focus. Dashed lines depict the exponential fits to each growth curve. **C.** Growth rate μ of simulated bacteria as a function of time within distinct regions of a simulated bacterial focus, averaged over 10 simulated foci. For B. and C., the color of the curves indicates the region within which bacterial growth is monitored, and the error bars depict the standard error of the mean.

bioRxiv preprint doi: https://doi.org/10.1101/2025.01.31.635960; this version posted February 1, 2025. The copyright holder for this preprint (which was not certified by peer review) is the author/funder, who has granted bioRxiv a license to display the preprint in perpetuity. It is made available under aCC-BY 4.0 International license.

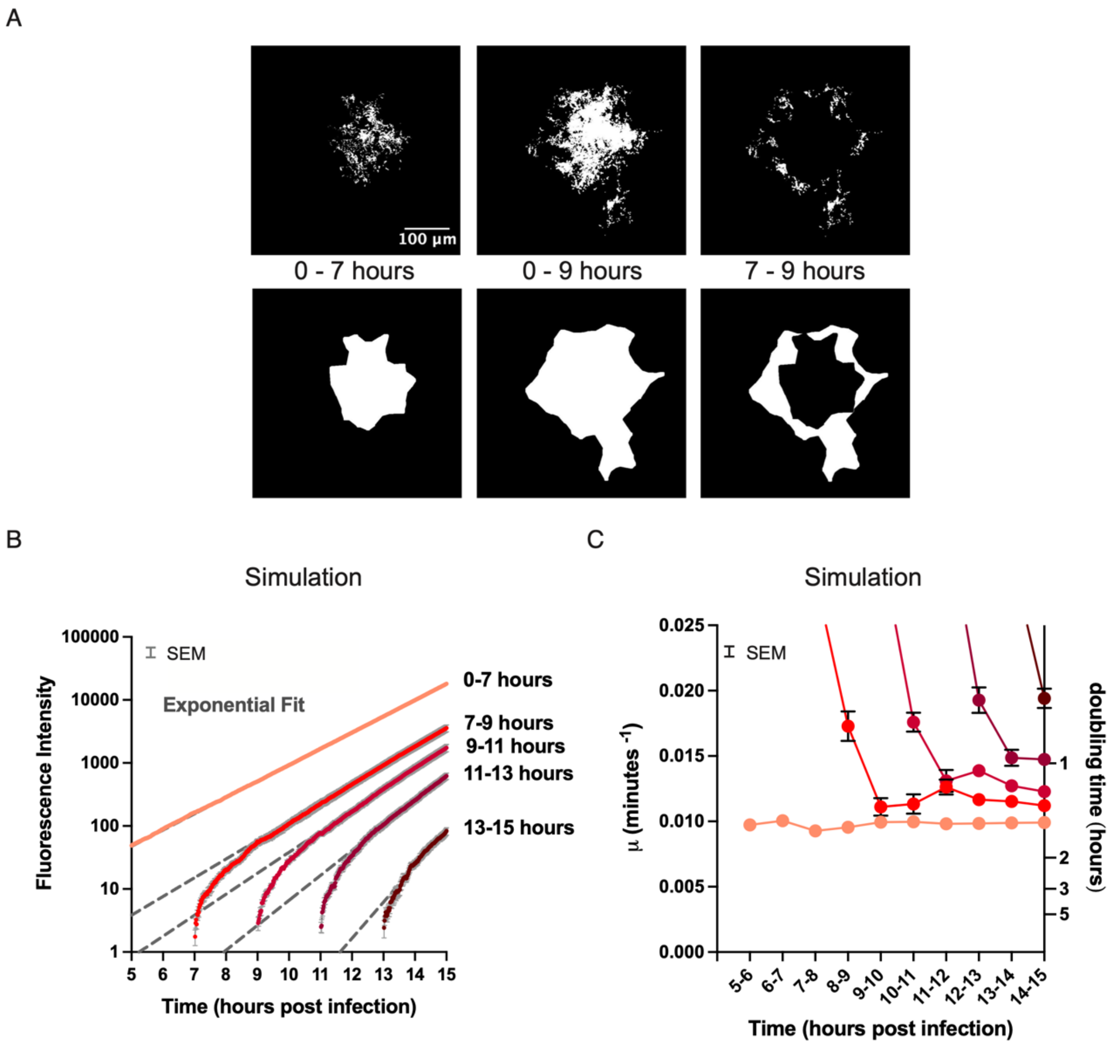

Supplemental Figure 2: Stochastic simulations of *Listeria monocytogenes* cell-to-cell spread aid in determining inflection point for bacterial growth within distinct regions of a bacterial focus.

bioRxiv preprint doi: https://doi.org/10.1101/2025.01.31.635960; this version posted February 1, 2025. The copyright holder for this preprint (which was not certified by peer review) is the author/funder, who has granted bioRxiv a license to display the preprint in perpetuity. It is made available under aCC-BY 4.0 International license.

Supplemental Figure 3: Quorum sensing among bacteria does not contribute to the decrease in growth rate of *Listeria monocytogenes* over time.

Growth rate as a function of time for cells WT *L. monocytogenes* EGD-e (light blue trace) and *ΔagrD L. monocytogenes* EGD-e (brown trace). The number of foci imaged for each condition include 17 and 10 foci respectively. P values were calculated using the Wilcoxon Rank-Sum test and denoted as asterisks above each data point. The error bars represent the standard error of the mean and the shaded region the standard deviation.

bioRxiv preprint doi: https://doi.org/10.1101/2025.01.31.635960; this version posted February 1, 2025. The copyright holder for this preprint (which was not certified by peer review) is the author/funder, who has granted bioRxiv a license to display the preprint in perpetuity. It is made available under aCC-BY 4.0 International license.

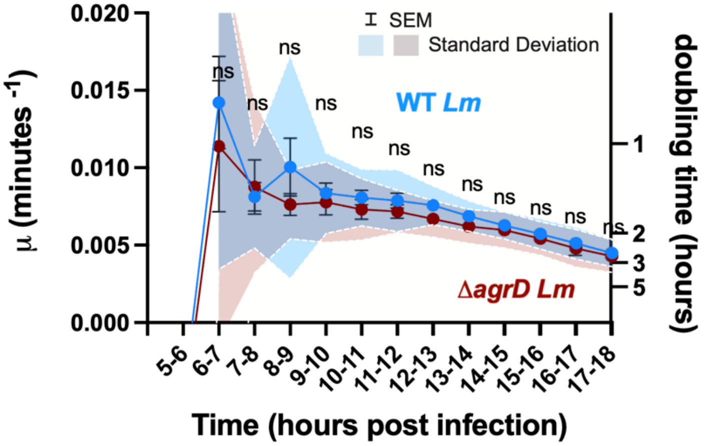

Supplemental Figure 3: Quorum sensing among bacteria does not contribute to the decrease in growth rate of *Listeria monocytognes* over time.

bioRxiv preprint doi: https://doi.org/10.1101/2025.01.31.635960; this version posted February 1, 2025. The copyright holder for this preprint (which was not certified by peer review) is the author/funder, who has granted bioRxiv a license to display the preprint in perpetuity. It is made available under aCC-BY 4.0 International license.

Supplemental Figure 4: Removal of the cytoplasmic domain of host cell E-cadherin reduces *L. monocytogenes* cell-to-cell spread and causes the growth rate inflection point to occur at an earlier time.

A. Growth rate μ as a function of time for bacteria propagating through WT E-cad (blue trace) and Δcyto E-cad (green trace) A431D cells. P values were calculated using the Wilcoxon Rank-Sum test and denoted as asterisks above each data point. Error bars represent the standard error of the mean and shaded region the standard deviation. 49 and 45 foci were imaged for each condition respectively. **B.** Comparison between inflection point T_i for bacteria replicating in WT E-cad or and Δcyto E-cad cells. P values were determined using the linear mixed-effects model. **C**. Inflection point at which bacterial growth begins to slow in each region of the focus in WT or Δcyto E-cad A431D cells. The color of each bar corresponds to the region on the schematics in Figure 1D. P-values were calculated using the Wilcoxon rank-sum test. 15 foci were included in the analysis for each condition.

bioRxiv preprint doi: https://doi.org/10.1101/2025.01.31.635960; this version posted February 1, 2025. The copyright holder for this preprint (which was not certified by peer review) is the author/funder, who has granted bioRxiv a license to display the preprint in perpetuity. It is made available under aCC-BY 4.0 International license.

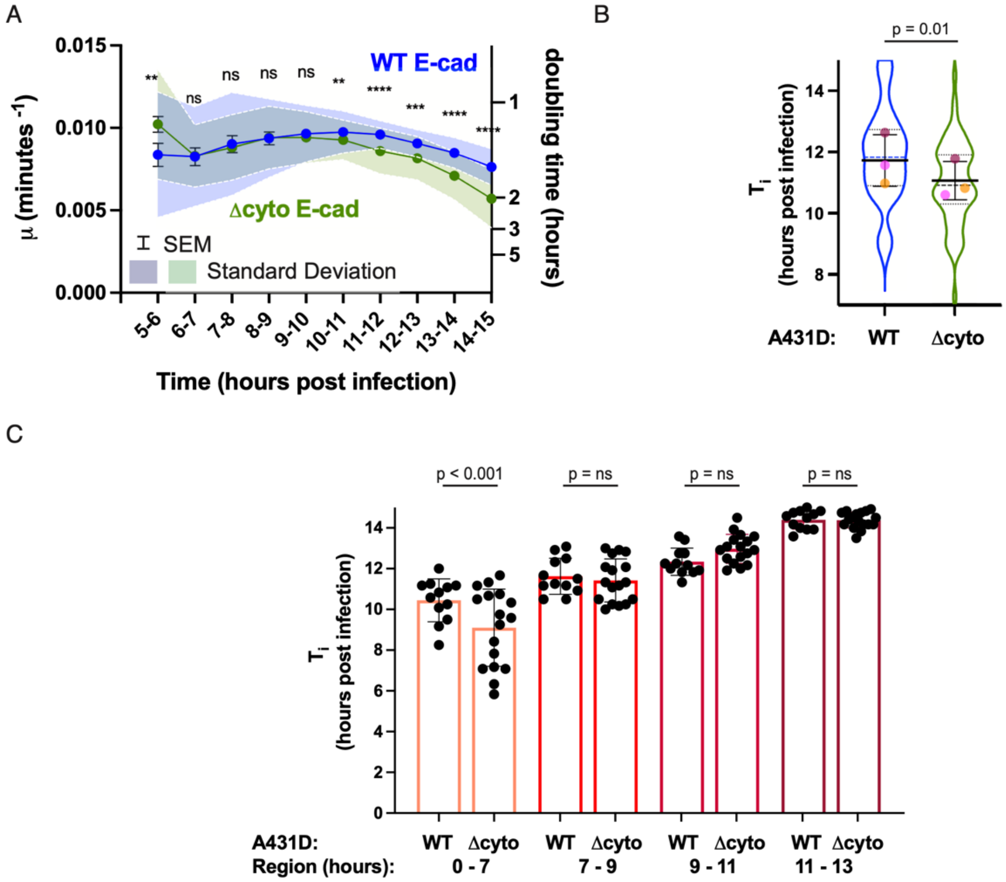

Supplemental Figure 4: Removal of the cytoplasmic domain of host cell E-cadherin reduces *L. monocytogenes* cell-to-cell spread and causes the growth rate inflection point to occur at an earlier time.
